# Biocompatibility and Osseointegration of Calcium Phosphate-Coated and Non-Coated Titanium Implants with Various Porosities

**DOI:** 10.17691/stm2021.13.2.06

**Published:** 2021-01-01

**Authors:** A.A. Korytkin, N.Yu. Orlinskaya, Ya.S. Novikova, S.A. Gerasimov, D.V. Davydenko, K.V. Kulakova, S.I. Tverdokhlebov, E.N. Bolbasov

**Affiliations:** Director, Novosibirsk Scientific Research Institute of Traumatology and Orthopedics named after Ya.L. Tsivyan of the Ministry of Health of the Russian Federation, 17 Frunze St., Novosibirsk, 630091, Russia; Professor, Head of Department of Pathological Anatomy with Tissue Conservation, University Clinic, Privolzhsky Research Medical University, 10/1 Minin and Pozharsky Square, Nizhny Novgorod, 603005, Russia; Chief Researcher, University Clinic, Privolzhsky Research Medical University, 10/1 Minin and Pozharsky Square, Nizhny Novgorod, 603005, Russia; Junior Researcher, Scientific Research Department, Novosibirsk Scientific Research Institute of Traumatology and Orthopedics named after Ya.L. Tsivyan of the Ministry of Health of the Russian Federation, 17 Frunze St., Novosibirsk, 630091, Russia; Head of Adult Orthopedics Department, University Clinic, Privolzhsky Research Medical University, 10/1 Minin and Pozharsky Square, Nizhny Novgorod, 603005, Russia; Researcher, Department of Pathological Anatomy with Tissue Conservation, University Clinic, Privolzhsky Research Medical University, 10/1 Minin and Pozharsky Square, Nizhny Novgorod, 603005, Russia; Researcher, Department of Pathological Anatomy with Tissue Conservation, University Clinic, Privolzhsky Research Medical University, 10/1 Minin and Pozharsky Square, Nizhny Novgorod, 603005, Russia; Acting Head of the Laboratory for Plasma Hybrid Systems, National Research Tomsk Polytechnic University, 30 Prospect Lenina, Tomsk, 634050, Russia; Researcher, Laboratory for Plasma Hybrid Systems, National Research Tomsk Polytechnic University, 30 Prospect Lenina, Tomsk, 634050, Russia

**Keywords:** osseointegration, porous titanium implants, cytotoxicity, bone defect, additive technologies, 3D printing

## Abstract

**The aim of the investigation** was to study the influence of pore size and the presence of a biologically active calcium phosphate coating in porous 3D printed titanium implants on the process of integration with the bone tissue.

**Materials and Methods:**

Samples of cylindrical implants with three different pore diameters (100, 200, and 400 μm) were fabricated from titanium powder on the Arcam 3D printer (Sweden) using electron beam melting technology. A calcium phosphate coating with a thickness of 20±4 μm was applied to some of the products by microarc oxidation. Cytotoxicity of the implants was determined *in vitro* on human dermal fibroblast cultures. The samples were implanted in the femoral bones of 36 rabbits *in vivo*. The animals were divided into 6 groups according to the bone implant samples. The prepared samples and peri-implant tissues were studied on days 90 and 180 after implantation using scanning electron microscopy and histological methods.

**Results:**

All samples under study were found to be non-toxic and well biocompatible with the bone tissue. There were revealed no differences between coated and non-coated implants of 100 and 200 μm pore diameters in terms of their histological structure, intensity of vascularization in the early stages, and bone formation in the later stages. Samples with pore diameters of 100 and 200 μm were easily removed from the bone tissue, the depth of bone growth into the pores of the implant was lower than in the samples with pore diameter of 400 μm (p<0.001). There were differences between coated and non-coated samples of 400 μm pore diameter, which was expressed in a more intensive osseointegration of samples with calcium phosphate coating (p<0.05).

**Conclusion:**

The optimal surface characteristics of the material for repairing bone defects are a pore diameter of 400 μm and the presence of a calcium phosphate coating.

## Introduction

Fabrication of joint implants with the use of additive technologies makes it possible to create individual products of complex shapes that combine high physical, mechanical, medical, and biological properties. The development of additive technologies provides great opportunities for practicing orthopedists and traumatologists to restore the function of damaged bone tissue [1].

Titanium has high strength and good biological compatibility with tissues during implantation [2, 3]. Individual implants made of titanium and its alloys by 3D printing allow repairing complex bone defects as they have overall open porosity and offer the possibility to regulate the size and shape of pores during the manufacturing process [2–6]. Experimental repair of a unicortical diaphyseal femoral defect in animals showed high biodegradability and osteoconductive properties of 3D porous material based on calcium phosphate [7]. However, products entirely made of tricalcium phosphate-based material have insufficient strength properties and are unsuitable as joint implants.

Calcium phosphate coatings formed by microarc oxidation on the surface of titanium implants are known to significantly improve the osteoinductive properties of products used for extraosseous and transosseous osteosynthesis [8, 9]. However, application of such 3D printed implants is limited in general clinical practice. The process of implant integration with the bone tissue and its osteoinductive and osteoconductive properties were found to be largely determined by the pore size and chemical composition of the implant surface [10–13]. Regulation of these parameters makes it possible to control the process of tissue histogenesis. However, the influence of pore size and calcium phosphate coating of individual titanium implants on their ability to integrate with bone tissue is still open to question. Solution to this problem will help to determine the characteristics of the material for manufacturing implants with the most optimal properties, which will permit achieving strong primary and subsequent secondary fixation.

**The aim of the investigation** was to study the influence of pore size and the presence of a biologically active calcium phosphate coating in porous 3D printed titanium implants on the process of integration with the bone tissue.

## Materials and Methods

Samples of cylindrical implants with three different pore diameters (100, 200, and 400 μm) were fabricated from titanium powder on the Arcam 3D printer (Sweden) using electron beam melting technology. All samples had an ordered hexagonal structure with outer strata located along the implant axis. To prevent scratch-fit effects when fixing the implants, they were fabricated without an aggressive rough coating on the surface. A calcium phosphate coating of 20±4 μm thickness was applied to some of the samples by the microarc oxidation method [8] using an experimental facility at the National Research Tomsk Polytechnic University (Russia). Next, wet-steam sterilization was carried out at a temperature of 134°C for 5 min in accordance with GOST R ISO 17665-1, which was followed by placing the samples in a triple sterile package. Cytotoxicity was studied according to GOST R ISO 10993.5 on an *in vitro* model (human diploid fibroblasts served as test culture).

*In vivo* studies were carried out on 36 male Soviet chinchilla rabbits aged 6±1 months with a body weight of 4675±258 g at the Department of Experimental Medicine at a vivarium, Privolzhsky Research Medical University (Nizhny Novgorod, Russia). The experiment was approved by the Ethics Committee of Privolzhsky Research Medical University.

The animals were divided into 6 groups according to the samples of bone implants (Table 1). The diameter of the sample base was 3.2 mm, the height — 8 mm.

**Table 1:** Characteristics of implant samples

Implant sample number	Pore diameter (μm)	Baseline porosity (%)	Calcium phosphate coating	Number of implants (pcs.)
1	100	55	+	6
2	100	55	—	6
3	200	62	+	6
4	200	62	—	6
5	400	70	+	6
6	400	70	—	6

According to the generally accepted ethical standards (Guide for the Care and Use of Laboratory Animals, National Research Council, 2011) and the ethical principles established by the European Convention for the Protection of Vertebrate Animals used for Experimental and Other Scientific Purposes (Strasbourg, 2006), all painful procedures were performed under general and local anesthesia.

After treatment of the operating field with antiseptic agents, the skin incision was performed in the projection of the lateral femoral condyle and soft tissues were divided. Using a drill with a bur diameter of 3 mm, a standardized cylindrical bone defect corresponding to the size of the implant sample was formed perpendicular to the femoral axis. After a bone sample was implanted into the formed defect, the surgical wound was sanitized and sutured layer by layer. X-ray control was performed using a C-arm system (portable X-ray diagnostic surgical device RTS-612 v. 4.2, Russia). In the postoperative period, dressings were changed and the operating wound was monitored for 10 days. The animals were removed from the experiment 90 and 180 days after the implantation, femoral bone areas were harvested in the area of direct contact with the implant samples.

Further preparation of the experimental materials for studies was carried out using equipment manufactured by Thermo Fisher Scientific (USA). The femur specimens were fixed in 10% formalin and decalcified, after that the implant samples were removed carefully from the bone tissue and taken for electron microscopy, and the bone tissue underwent standard histologic examination using an Excelsior ES apparatus. Next, the samples were embedded in paraffin blocks using the HistoStar embedding workstation. Serial 4–6   μm-thick sections were obtained on a Microm HM 325 microtome. The sections were stained with hematoxylin and eosin using a Gemini AS staining station.

A Leica 2500 microscope (Leica Microsystems, Germany) was used for morphometric processing. The morphological changes in the bone tissue were assessed via histological examination.

Electron microscopic studies of implant samples were carried out at Research Equipment Sharing Center “New materials and resource-saving technologies”, National Research Lobachevsky State University of Nizhni Novgorod (Russia), using a JSM-IT300LV scanning electron microscope (JEOL, Japan) in high vacuum mode at low probe currents (<0.1 nA) to reduce the effect of the electron beam on the studied samples (electron probe diameter up to 3 nm). Photo documenting was carried out at magnifications from 27–30 (for overview images) to 2500–3700 (for evaluating the surface of coated and non-coated implants, as well as pores and individual informative inclusions in the surface structure of samples under study).

Microscopic examination of the implant samples involved semi-quantitative morphometric evaluation of bone tissue changes using a four-score assessment scale. When evaluating inflammatory response, the specimens were assigned scores from 0 (no signs of inflammation) to 3 (significant inflammation). Vascularization was assessed with scores from 0 (no signs) to 3 (significant vascularization); osteogenesis intensity — from 0 (no signs of osteogenesis) to 3 (intense osteogenesis, a large area is occupied by mature bone tissue); sclerosis — from 0 (no signs) to 3 (severe sclerosis). The depth of bone tissue growth into the implant pores was expressed in relative units: 0 — no growth, 1 — weak growth, 2 — moderate growth, 3 — profound growth.

### Statistical analysis

Statistical data processing was performed using Microsoft Office Excel and Statistica 6.1. The results of the study were assessed using nonparametric statistical methods: the Mann–Whitney U-test was used for paired comparisons, the Kruskal–Wallis test — for multiple comparisons. Differences between the compared groups were considered statistically significant at p<0.05.

## Results and Discussion

The absence of the cytotoxic effect of the material used for sample manufacturing was established on an *in vitro* model. The results of *in vivo* studies showed that in most of the experimental animals all the studied parameters, including the general condition (weight, hair, skin, etc.), hematological and biochemical parameters, were within the normal range on days 90 and 180 after implantation of the samples.

Semi-quantitative morphometric assessment of changes in bone tissue was carried out during microscopic examination of the implant samples, the results are presented in Table 2.

**Table 2:** Semi-quantitative morphometric assessment of changes in bone tissue at different time periods after implantation of samples (scores)

Implant sample number	Inflammation	Vascularization	Osteogenesis	Sclerosis	Depth of bone tissue growth
***Day 90***					
1	0	2	1	0	1
2	0	2	1	1*	1
3	0	2	1	1*	1
4	0	1	1	1*	1
5	0	3*^#^	2*^#^	0	2*
6	0	2	1	0	2*
***Day 180***					
1	0	1	2	0	2
2	0	1	2	0	2
3	0	1	2	1*	2
4	0	1	2	1*	2
5	0	0	3*^#^	0	3*
6	0	0	2	0	3*

* p<0.001 — differences between groups, Kruskal–Wallis test; ^#^ p<0.05 — differences between groups when comparing groups with the same pore diameter with or without coating, Mann–Whitney test.

**Figure 1:**
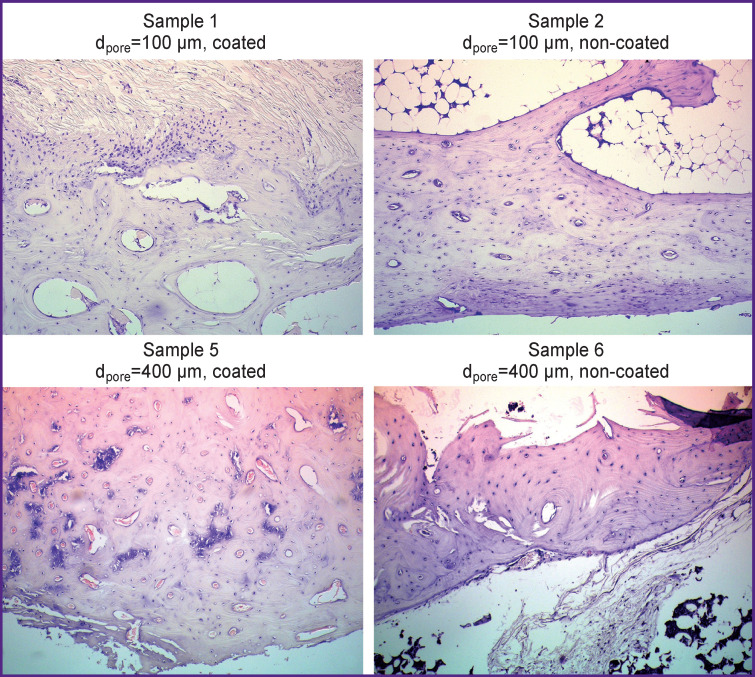
Histological picture of peri-implant tissues 180 days after implantation of samples Staining with hematoxylin and eosin, ×200

Histological examination showed that after implantation of the samples, there were no signs of inflammation of the peri-implant tissues in any case. In groups 5 and 6, mature bone trabeculae were observed along the periphery of the implantation area, their prevalence was higher than with samples 1–4 (p<0.05). Sclerosis was less significant after implantation of samples 1, 2 while being insignificant in samples 5, 6 (p<0.001) (Figure 1).

**Figure 2:**
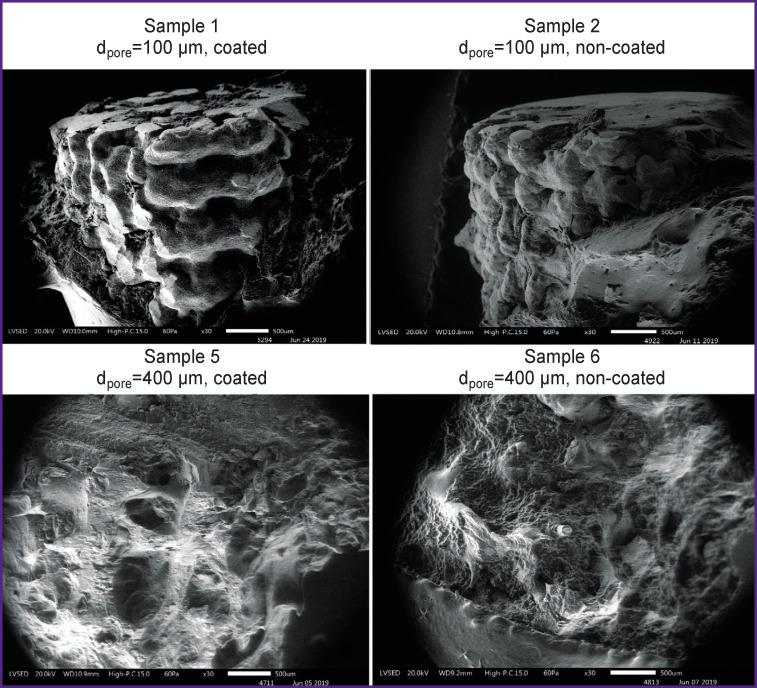
Overall view of implant samples 180 days after implantation The presence of a massive layer of newly formed bone on the implant surface; electron microscopy, ×30

Electron microscopic examination of the samples removed 90 days after the implantation showed that pores were filled with bone structures of varying degrees of maturity over the entire area in groups 1 and 2, but the depth of their filling was somewhat lower than in groups 3–6. Mature trabeculae with clearly defined osteocytic pattern commonly found in a mature structure occupied a larger part (p<0.001) of the implantation area in groups 5, 6 as compared to other groups. After 180 days of the study, the area occupied by the newly formed mature bone tissue in the pores of the implants increased: the increase in the area was greater in groups 5, 6 as compared to the implants from groups 1–4 (p<0.001). Overview electron microscopic images and the surface of implant samples are shown in Figure 2.

Comparative analysis of implants showed that the presence or absence of a coating makes no matter in samples with pore diameters of 100 and 200 μm since there were no differences revealed in the histological structure, intensity of vascularization in the early stages, and bone formation in the later stages. Samples with pore diameters of 100 and 200 μm could be easily removed from the bone tissue at all periods of the study, since the depth of bone growth into the implant pores was lower than in samples 5 and 6 (p<0.001). There was a difference observed between samples 5 and 6 at a pore diameter of 400 μm during different periods of the study, which was exhibited in a more intensive osseointegration in sample 5 that had a coating (p<0.001 and p<0.05 90 and 180 days after implantation, respectively).

Many authors agree when it comes to biodegradable calcium phosphate coatings of titanium implants: coatings significantly improve their osteoinductive properties and promote osteogenesis [6, 8, 9, 14]. Kalinichenko et al. [14] studied how a biodegradable calcium phosphate and hydroxyapatite coating applied to titanium implants by plasma electrolytic oxidation affected osteogenesis in the setting of simulated closed fracture of the rat femur. It was found that biodegradable implant coatings promoted proliferation and expression of the BMP-2, VEGF, and TGF-β2 genes and enhanced the regulatory effects of growth factors at different stages of reparative osteogenesis. The authors also concluded that under conditions of biomechanical loads, topography of the implant surface promoted adhesion and retention of osteoblasts on it. Calcium phosphates are a source of biologically active ions that create a favorable chemical environment for osteogenic cells and ensure stability of proliferation of newly formed bone tissue cells.

We have demonstrated that the presence of a calcium phosphate coating does not always improve osteogenesis: presence or absence of coatings makes no difference in implants with pore diameters of 100 and 200 μm. However, the presence of a coating promotes more intensive osseointegration when a pore diameter is 400 μm.

Researchers have different opinions regarding the optimal pore size for better osseointegration. Tikhilov et al. [5] investigated bone and soft tissue integration of porous titanium implants with a pore size of 100–200 μm. Such products promoted bone ingrowth to a depth of 2–3 mm and provided good fixation of the implant. The authors consider this pore diameter to be optimal for the integration of both bone tissue and soft tissues.

Another group of researchers [15] found that among 3D printed porous titanium implants they studied, osteogenesis indicators of implants with the actual pore size of about 600 μm were superior to those of two other groups with pore sizes of 400 and 800 μm.

Some authors have drawn the conclusion that the larger the pore size, the better osseointegration occurs. Zhao et al. [16] have found that polylactide scaffolds with large pore sizes show the best results in bone tissue regeneration. A large pore size (2 mm) is more favorable for the exchange of cells and cytokines between the periosteum and cortical bone. Besides, osteogenesis and angiogenesis are two interrelated processes, and viability of the formed bone tissue depends on regeneration of new vessels. The authors have shown that vascularization of the new bone increases with an increase in pore size. However, mechanical properties of polylactide scaffolds were found to decline with increasing pore diameter. In our case, the use of titanium alloy implants prevents such disadvantage as low strength.

Ilea et al. [17] showed in their study that titanium scaffolds with pores of 800 nm in diameter exhibited better osseointegration than scaffolds with pores of 1000 nm. Similar results were obtained by Liu et al. [18]. The authors studied osteogenesis of 3D printed calcium phosphate bone scaffolds made with 70% porosity and a large pore size of 0.8, 1.2, and 1.6 mm. The scaffolds were implanted into an 8 mm defect created in the cranial vault of New Zealand rabbits and examined 4 and 8 weeks after the implantation. All the scaffolds showed excellent mechanical properties and had better bone-forming ability than the control, both after 4 and 8 weeks. Of all the scaffolds, those with pore size of 800 μm appeared to be superior to the others in all bone formation parameters.

The likely reason why the results of research on the optimal pore size for osseointegration differ is that the authors represent the average pore size without taking into account their interrelation, overall porosity. Therefore, no consensus has been achieved on the most optimal surface characteristics of materials for manufacturing bone implants.

## Conclusion

Highly porous titanium implants manufactured by using additive technologies are promising for reconstructive and revision surgery in repairing bone defects. The studied implant samples have high biocompatibility, no cytotoxic effect. The most optimal surface characteristics of implant sample material promoting osseointegration are pore diameter of 400 μm and the presence of a calcium phosphate coating.

## References

[1] Eltorai A.E.M., Nguyen E., Daniels A.H. (2015). Three-dimensional printing in orthopedic surgery.. Orthopedics.

[2] Rosenberg O.A., Sheikin S.E., Sochan S.V. (2010). Prospects for the using of technically pure titanium for implants in bone surgery.. NMT.

[3] Bondarenko S., Dedukh N., Filipenko V., Akonjom M., Badnaoui A.A., Schwarzkopf R. (2018). Comparative analysis of osseointegration in various types of acetabular implant materials.. Hip Int.

[4] Korytkin A.A., Zakharova D.V., Novikova Ya.S., Gorbatov R.O., Kovaldov K.A., El Moudni Y.M. (2017). Custom triflange acetabular components in revision hip replacement (experience review).. Travmatologiya i ortopediya Rossii.

[5] Tikhilov R.M., Shubnyakov I.I., Denisov A.O., Konev V.A., Gofman I.V., Mikhailova P.M., Netylko G.I., Vasiliev A.V., Anisimova L.O., Bilyk S.S. (2018). Bone and soft tissues integration in porous titanium implants (experimental research).. Travmatologiya i ortopediya Rossii.

[6] Qadir M., Li Y., Munir K., Wen C. (2018). Calcium phosphate-based composite coating by micro-arc oxidation (MAO) for biomedical application: a review.. Crit Rev Solid State.

[7] Dubrov V.E., Klimashina E.S., Shcherbakov I.M., Shipunov G.A., Putlyaev V.I., Evdokimov P.V., Tikhonov A.A., Zyuzin D.A., Danilova N.V., Mal’kov P.G. (2019). Experimental evaluation of the properties of 3D porous bone substitute based on calcium phosphate on the model of monocortical diaphysial femur defect in rats.. Bull Exp Biol Med.

[8] Bolbasov E.N., Popkov D.A., Kononovich N.A., Gorbach E.N., Khlusov I.A., Golovkin A.S., Stankevich K.S., Ignatov V.P., Bouznik V.M., Anissimov Y.G., Tverdokhlebov S.I., Popkov A.V. (2019). Flexible intramedullary nails for limb lengthening: a comprehensive comparative study of three nails types.. Biomed Mater.

[9] Popkov A.V., Popkov D.A., Kononovich N.A., Gorbach E.N., Tverdokhlebov S.I., Bolbasov E.N., Darvin E.O. (2018). Biological activity of the implant for internal fixation.. J Tissue Eng Regen Med.

[10] Albrektsson T., Johansson C. (2001). Osteoinduction osteoconduction and osseointegration.. Eur Spine J.

[11] Gittens R.A., Olivares-Navarrete R., Schwartz Z., Boyan B.D. (2014). Implant osseointegration and the role of microroughness and nanostructures: lessons for spine implants.. Acta Biomater.

[12] Hayes J.S., Klöppel H., Wieling R., Sprecher C.M., Richards R.G. (2017). Influence of steel implant surface microtopography on soft and hard tissue integration.. J Biomed Mater Res B Appl Biomater.

[13] Tavakoli J., Khosroshahi M.E. (2018). Surface morphology characterization of laser-induced titanium implants: lesson to enhance osseointegration process.. Biomed Eng Lett.

[14] Kalinichenko S.G., Matveeva N.Y., Kostiv R.Y., Edranov S.S. (2019). The topography and proliferative activity of cells immunoreactive to various growth factors in rat femoral bone tissues after experimental fracture and implantation of titanium implants with bioactive biodegradable coatings.. Biomed Mater Eng.

[15] Ran Q., Yang W., Hu Y., Shen X., Yu Y., Xiang Y., Cai K. (2018). Osteogenesis of 3D printed porous Ti6Al4V implants with different pore sizes.. J Mech Behav Biomed Mater.

[16] Zhao D., Jiang W., Wang Y., Wang C., Zhang X., Li Q., Han D. (2020). Three-dimensional-printed poly-l-lactic acid scaffolds with different pore sizes influence periosteal distraction osteogenesis of a rabbit skull.. Biomed Res Int.

[17] Ilea A., Vrabie O.G., Băbţan A.M., Miclăuş V., Ruxanda F., Sárközi M., Barbu-Tudoran L., Mager V., Berce C., Boşca B.A., Petrescu N.B., Cadar O., Câmpian R.S., Barabás R. (2019). Osseointegration of titanium scaffolds manufactured by selective laser melting in rabbit femur defect model.. J Mater Sci Mater Med.

[18] Liu F., Liu Y., Li X., Wang X., Li D., Chung S., Chen C., Lee I.S. (2019). Osteogenesis of 3D printed macro-pore size biphasic calcium phosphate scaffold in rabbit calvaria.. J Biomater Appl.

